# Macrophage migration inhibitory factor: a potential driver and biomarker for head and neck squamous cell carcinoma

**DOI:** 10.18632/oncotarget.12890

**Published:** 2016-10-25

**Authors:** Sha-sha Wang, Xiao Cen, Xin-hua Liang, Ya-ling Tang

**Affiliations:** ^1^ State Key Laboratory of Oral Diseases, West China Hospital of Stomatology, Sichuan University, Chengdu Sichuan, People’s Republic of China; ^2^ Department of Oral and Maxillofacial Surgery, West China College of Stomatology, Sichuan University, Chengdu Sichuan, People’s Republic of China; ^3^ Department of Oral Pathology, West China Hospital of Stomatology, Sichuan University, Chengdu Sichuan, People’s Republic of China

**Keywords:** macrophage migration inhibitory factor, head and neck squamous cell carcinoma, tumorigenesis, metastasis, biomarker

## Abstract

Macrophage migration inhibitory factor (MIF), a pleiotropic proinflammatory cytokine, has been showed to be associated with the immunopathogenesis of many diseases. Recent study demonstrated that MIF promoted tumorigenesis and tumor progression and played a critical role in various kinds of human cancer including head and neck squamous cell carcinoma(HNSCC). Hence, in this paper we retrospected the relationship between MIF and angiogenesis, epithelial-mesenchymal transition (EMT), inflammation, immune response, hypoxia microenvironment, and discussed whether it is a promising biomarker for diagnosis and supervisor of HNSCC.

## INTRODUCTION

Head and neck squamous cell carcinoma (HNSCC), which accounts for 90 % of head and neck cancers, has been reported as the sixth common cancer worldwide and the third most common cancer in developing nations. [1–3] Despite substantial advances in conventional treatment including surgery, radiotherapy, chemotherapy for HNSCC patients in recent decades, HNSCC continues to remain a dismal prognosis, of which the five-year overall and disease-free survival is only about 50%. [4] One of the main reasons is that HNSCC patients are often diagnosed at advanced stages. For the insufficiency of efficaciously therapeutic modalities, patients generally suffer from severe and debilitating adverse effects resulting from surgery and chemoradiotherapy even they are successfully cured. Thus, there is an obvious need for new biomarkers to diagnose HNSCC at an early stage or even provide alternative targeted therapeutic strategies.

Macrophage migration inhibitory factor (MIF), a T-cell-derived factor, was firstly thought to inhibit the migration of macrophages in experiments designed to characterize delayed-type hypersensitivity and hence derived its name in 1966. [5] Later, the molecule was verified secretion by a variety of cells including eosinophils, [6] lymphocytes, [7] and macrophages. [8] Extensive studies conducted on MIF revealed that it primarily acted as a proinflammatory protein. [9] The close association between MIF and innate immunity was first apparently revealed by studies of endotoxic shock models. [10] In 1996, the inhibition of T-cell activation and antibody production by MIF formed its relation with adaptive immunity. [7] Subsequently, many investigators demonstrated the central role of MIF in cancer-associated immune response. [11] For its pleiotropic effects on normally cellular activities, inflammatory and immune processes, there was enough evidence to confirm that MIF was capable of providing several levels of support to a developing tumor. Recently, a series of studies have enlightened that MIF governed angiogenesis, [12] epithelial-mesenchymal transition (EMT), [13] hypoxia [14] and cell cycle [12] in many kinds of human cancers including HNSCC, and indicated that MIF might be a potential driver and biomarker for HNSCC.

## MIF'S GENE AND STRUCTURE

The *MIF* lies on the human genome (22q11.2), regulated by the two polymorphic sites (CATT repeat at -794 and a single nucleotide polymorphism at -173 (G/C)) in the promoter region. [15–17] Though the exonic structure or the sequence of *MIF* is highly conserved across phylogeny, there is a remarkable feature of the human *MIF* gene that is the presence of a microsatellite repeat (CATT)_5-8_ within the 5′ promoter region. [16] It is associated with plasma MIF level, severity of inflammatory diseases, and risk of cancer. [18] A detailed study revealed the individuals carrying five-CATT allele displayed lowest MIF level, while those with the six-, seven-, and eight-CATT alleles showed proportionally increased gene expression. [16]

MIF is a molecule comprised of 115 amino acids with a molecular weight of 12.5 kDa. [19] In the active form, MIF is aligned by three 114-residue monomers to form a symmetrical trimer, the catalytic active site located between two adjacent monomers of the homotrimer, which has a strong homology with the enzyme D -dopachrome-tautomerase (DDT). [20, 21] (*S,R*)-3-(4-hydroxyphenyl)-4,5-dihydro-5-

isoxazole acetic acid methyl ester (ISO-1), an inhibitor of DDT, could decrease wild-type or mutant MIF activity in human and murine mononuclear cells. So there was a hypothesis that MIF also displayed some enzymatic activity. [22]

## MIF AND ASSOCIATED SIGNALING PATHWAYS

Although MIF was found in the 1960s, the cell surface receptor for MIF was identified until more than 35 years later. [23] CD74 was identified as a high affinity cell surface binding protein for extracellular MIF with the help of expression cloning and functional analyses. [24] The prostate cancer invasion could be attenuated by the inhibition of MIF or CD74. [25] CD74 is a nonpolymorphic type II integral transmembrane protein which is involved in the transport from the Golgi apparatus to the endoplasmic reticulum. It is abundant on the cell surface, expressed on monocytes/macrophages, B cells and mesenchymal, epithelial and endothelial cells. [24] Kindt et al. proved that MIF/CD74 pathway was involved in the HNSCC progression. Knockdown of CD74 could slow down the proliferation and invasiveness of a squamous carcinoma cell line SCCVII as well as negatively affected the growth of orthotopic tumors generated by SCCVII cell inoculation. [12] The complex formed by MIF binding to the extracellular C-terminal domain of CD74 initiates MIF-dependent sustained activation of the ERK1/2 MAPK cascade resulting in increased cell proliferation *via* cyclin D1 transcription and subsequent phosphorylation of the Rb gene and prostaglandin E2 production. [23, 26] There are another pathways associated with the MIF-dependent activation of this cascade. Jab-1/CSN5, a protein that serves as an intracellular binding partner of MIF, and Src tyrosine kinase signaling pathway are involved in fast and transient activation of the ERK MAPK signalling pathway. [27] Besides, CD44, a transmembrane coreceptor, is required in the MIF-mediated ERK1/2 kinase phosphorylation for its role in serine phosphorylation. [28] The long-term enhanced activation of CD44 heightened inflammatory response and was responsible for promotion cancer invasion. [29] Intriguingly, MIF is also described as a non-cognate ligand for CXCR2 and CXCR4 which are ascribed to functional receptors. [30] In this context, MIF has to compete with known cognate ligands to bind with these receptors. MIF/CXCR2 interaction was identified to elicit the recruitment and arrest of monocytes, [31] whereas MIF-mediated T-cell recruitment was traced to the interaction of MIF and CXCR4. [30]

The nuclear factor - kappa B (NF-κB) which plays a vital role in carcinogenesis is involved in the MIF associated signaling pathways. In nasopharyngeal carcinoma (NPC) cell lines, C666-1, MIF/IL-8/ CXCR2 signaling could enhance the growth of the tumor spheres. And NF-κB inhibitor parthenolide could inhibit the gene expression of IL-8. [32] Lv et al. found that MIF assisted lung metastasis of breast cancer *via* activation of HMGB1/TLR4/NF-κB axis. [33] In the non-small cell lung cancer cell lines, the dissociation of MIF- ribosomal protein S3 complex induced by ionizing radiation sequentially activated NF-κB and made the expression of target genes of this factor, which promoted tumor metastatic conversion. [34]

## MIF AND INFLAMMATION MICRO-ENVIRONMENT

The notion of MIF as a proinflammatory protein was firstly confirmed by the studies about delayed hypersensitivity and further evaluated in the mouse model of septic shock. [10] It was involved in many aspects of inflammatory. The level of MIF was elevated in both serum and synovial fluid of patients with rheumatoid arthritis. The anti-inflammatory effect of steroids has been proved both *in vitro* and *in vivo*. It was shown that MIF secretion was inhibited by high anti-inflammatory concentration of steroids. [5] Compared to the control group, the mortality rate of MIF-knockout mice showed a significant reduce in response to lipopolysaccharide (LPS). [35] Bacher et al. noted that MIF also influenced the proliferation and activation of T cells. [7] As mentioned previously, MIF had a direct effect on macrophages. In turn, MIF secretion could be induced by LPS in the murine macrophage cell line. [8] Moreover, in response to tumor necrosis factor (TNF) and interferon, macrophages-released MIF was increased, and this led to increasing the production of NO and TNF-α in an autocrine fashion, resulting in the removal of bacteria from infected tissue. [36] Additionally, MIF reduced the rate of apoptosis in neutrophil granulocytes. Baumann et al. reported that MIF delayed apoptosis in neutrophils by inhibiting the mitochondria-dependent death pathway. [37]

The role of MIF in inflammation underlined its position in the development of HNSCC. In laryngeal carcinoma samples, Kindt et al. emphasized that the elevated MIF level was associated with a slightly decreased abundance of CD3^+^ T cells in the peritumoral tissue. [38] In another study, tumor-derived MIF could recruit neutrophils *via* CXCR2-dependent chemotaxis and trigger the inflammatory activity by eliciting neutrophils’ release of C-C Motif Ligand 4 (CCL4) and matrix metalloprotease 9 (MMP9) in human hypopharyngeal carcinoma cell line FaDu. [39] Both CCL4 and MMP9 have been proved to be involved in several stages of tumor progression in other cancers. [40–42] Li et al. demonstrated that tumor-derived MIF promoted the generation and recruitment of Th17 cells dependent on the mTOR pathway and mediated by the MIF-CXCR4 axis in NPC. Th17 cells in tumor tissue produced more IFN-γ than healthy controls. Besides, the frequency of MIF-positive tumor-infiltrating lymphocytes in NPC tissue was positively correlated with patients clinical outcomes. [43] With observations that the high-expression of MIF and IL-8 was significantly associated with increased lymph node metastasis in NPC patients and exogenous MIF treatment alone could upregulate IL-8 secretion in CNE-1 and CNE-2 NPC cells *in vitro*, Liao et al. inferred that MIF contributed to lymph node metastasis by upregulating IL-8 expression. [44]

## MIF AND TUMOR HYPOXIA MICRO-ENVIRONMENT

The crosstalk between tumor cells and microenvironment of the host is an important driving force in the selection of clone that is prone to invasion and metastasis. Hypoxia, resulting from rapid tumor growth in the absence of accompanying blood supply, is a critical symbol and determinant of tumor microenvironment. To maintain growth advantage, tumor cells under hypoxia microenvironment enhanced expression of hypoxia-inducible factor-1 (HIF-1), which favors anaerobic tumor growth, resistance to therapy, and metastatic adaptation. [45] HIF-1, as a heterodimeric transcription factor, is composed of α and β subunits. HIF-1β is constitutively expressed whereas HIF-1α, the active subunit, is undetectable under normoxia for rapid proteasomal degradation. [46] Arguably, a series of facts have indicated that hypoxia is a potent inducer of MIF secretion in kinds of cells. [47–49] Our group demonstrated that hypoxia stimulated the accumulation of CD11b^+^Gr-1^+^ myeloid cells by elevating production of MIF *via* HIF-1α/HIF-2α-dependent ways in HNSCC. [14] Fu et al. found that HIF-1α rapidly induced MIF expression in human vascular smooth muscle cells *via* ERK activity. [50] Baugh et al. showed that MIF was a direct HIF-1 transcriptional target. HIF-1 bound to the hypoxia response elements in the 5´UTR of the *MIF*. [51] Mladenova et al. demonstrated that HIF-1α knockout caused downregulation of MIF in a mouse model of proximal colon cancer. [52] Increasing evidence has indicated that cellular senescence serves as a tumor suppressor and that the host may utilize senescence as an anti-tumor defence mechanism. HIF-1α delayed premature senescence, and thus activation of HIF-1α in tumor cells would limit premature senescence and confer a biological advantage on these cells. Using embryonic fibroblasts from HIF- 1α knockout mouse, the onset of cellular senescence significantly accelerated and the cellular division decreased under hypoxia condition. Welford et al. identified that the MIF was a crucial effector of HIF-1α delaying senescence. [53]

Conversely, MIF also contributed to stabilize HIF-1. No et al. suggested that a ternary complex formed by HIF-1α, MIF, and CSN9 signalosome subunit 5 (the bridge between HIF-1α and MIF) was necessary to prevent degradation of HIF-1α under aerobic condition. [54] Oda et al. demonstrated that MIF enhanced activation of HIF-1 under hypoxic condition in MCF-7 cells *via* p53-dependent manner *in vitro* and *in vivo*. [55]

However, Larsen et al. found that inhibition of HIF-1α, HIF-2α using siRNA had no effect on hypoxia-induced MIF secretion in MCF-7 breast cancer cells, which hinted that there existed other hypoxia-induced regulatory mechanisms for the up-regulation of MIF. The authors considered that NF-κB and C/EBPβ signaling pathways were association with this phenomenon. [56]

## MIF AND ANGIOGENESIS

Analogous to normal tissues, tumors require vessels to sustainably supply nutrients and oxygen as well as evacuate metabolic wastes and carbon dioxide simultaneously. Indeed, no matter synthetic or catabolic metabolism is excessively exuberant in tumors. Hence, it is necessary to induce new blood vessels to meet tumor cells metabolism. Angiogenesis, as a vital mode to achieve this goal, is the formation of new blood vessels from the existing vasculature by sprouting. [57,58] Opposite to the orderly normal vessels, the organization of these vessels is tortuous, saccular, and chaotic. The structure of vessel wall is also abnormal, which is formed by endothelial cells with large gaps, detached pericytes and abnormal basement membranes. The excessively leaky vessels are prone to fuel the tumor metastasis. [59] Therefore, angiogenesis is regarded as one hallmark of cancer.

Accumulated evidence has proved that MIF was associated with tumor angiogenesis. When the B lymphoma mouse model was administrated with monoclonal antibody (mAb) specific for MIF, Chesney et al. observed a marked reduction in B lymphoma growth. However, profound analysis showed that anti-MIF antibody unaffected B-cell lymphoma proliferation. Reciprocally, microvascular endothelial cells were sensitivity to the mAb, whose proliferation was dependent on MIF. This result suggested that the antitumor effect caused by anti-MIF resulted from MIF suppression on angiogenesis. [11] Subsequently, Shimizu et al. reported that anti-MIF reduced xenografted melanoma-associated angiogenesis. [60] Further study showed that both the ERK and the PI3K pathways were regarded as the MIF-signaling pathways that underly this angiogenic response. [61]

Vascular endothelial growth factor (VEGF) is the well-known angiogenesis inducer. [62] One possible mechanism of MIF-induced angiogenesis in HNSCC was related to the upregulation of VEGF. When MIF/CD74 signaling pathway was blockaded *via* using anti-CD74 shRNA, the SCCVII cells showed a significant decrease in producing VEGF compared to the control, which damaged the capacity of promotion angiogenesis. [12] IL-8, another prominent pro-angiogenic molecule, was considered to associate with the angiogenesis in HNSCC. In NPC tissue, the high-expression of MIF and IL-8 was significantly associated with increased intratumoral microvessel density and microvessels or lymph node metastasis in NPC patients. Liao et al. inferred that MIF contributed to lymph node metastasis by inducing angiogenesis through the way of upregulating IL-8 expression. [44]

## MIF AND EMT

EMT is a process that polarized epithelial cells convert into motile mesenchymal phenotype which is characterized by suppression of the adherent protein E-cadherin and overexpression mesenchymal markers, such as N-cadherin and vimentin. [63–66] In this process, epithelial cells lose their cell-cell adhesion and acquire migratory and invasive properties, contributing to the gaining mesenchymal stem cell characteristics. [67, 68] A series of transcription factors, for instance, Snail, Slug, Twist, and Zeb1/2, which control the expression of proteins involved in cell polarity, cell-cell adhesion, cytoskeleton structure and extracellular matrix degradation, orchestrate the EMT and contribute to the initial invasion and metastatic dissemination of carcinoma cells. [69] The major development signaling circuits, including TGF-β/Smad, Wnt, and growth factor receptor signaling cascades, and their crosstalks have been implicated in some aspects of the EMT program. [70, 71] A compelling body of evidence has indicated that EMT is a crucial procedure in metastasis cascade for epithelium-derived cancer cells.

MIF has been deemed one of the factors triggering EMT. In A549 lung adenocarcinoma cells, Keshamouni et al. identified that MIF was up-regulated during TGF-β induced EMT with quantitative differential proteomic analysis. [72] Funamizu et al. found that the overexpression of MIF decreased E-cadherin and increased vimentin and ZEB1/2 in pancreatic cancer cells. And it was considered to be mediated by miR-200b, a member of miR-200 family. [73] When the colorectal cancer cells were cultured in conditioned media containing higher level of soluble MIF, the tumor cells elevated N-cadherin and vimentin expression and decreased E-cadherin expression. Although these findings have established the links between MIF and EMT, there still need to make further efforts to elucidate the exact mechanism. [74] Zeng et al. utilized small siRNA to knock down the expression of MIF which inhibited the proliferation, migration, and colony formation of oral squamous cell carcinoma (OSCC) cells. They also found that Twist1, the transcriptional factor of the EMT, downregulated concomitantly in the MIF-knockdown (KD) OSCC cells. [13] In breast cancer cell, along with enhancing MIF expression, the expression of snail, vimentin and twist was increased in a time-dependent manner. Conversely, the expression of snail, vimentin and twist could be decreased by the MIF knockdown. [33]

## MIF AND CELL PROLIFERATION, APOPTOSIS AND AUTOPHAGY

Research evidence supported that the activation of MIF was involved in cell proliferation and apoptosis. [75] During cell cycle, there are various check point proteins so that cells can correct the errors in DNA replication during proliferation and force the abnormal cells to undergo apoptosis. [76] As we known, the balance of proliferation and apoptosis is necessary for the normal cell development. MIF can interfere with the cell cycle check points so that cells proliferate at a very high rate. Li et al. showed that recombinant human MIF increased the proliferation of gastric cancer MGC-803 cells by inducing the expression of cyclin D1 and inhibiting the expression of p27^Kip1^ *via* the PI3K/Akt pathway. [77] Both the cyclin D1 and p27^Kip^ contributed to regulate cell cycle progression from G1 to S phase. [78] Thus, the abnormal expression of them led to the loss of control of cell proliferation. Wen et al. demonstrated that siRNA-mediated knockdown of MIF caused the downregulation of cyclin D1 and cyclin-dependent kinase 4 (CDK4) which activated cell progression from G1 to S phase. [79] In renal cell carcinoma, miRNA-451 inhibited cell proliferation, migration and invasion through up-regulation of MIF. [80] SiRNA against MIF significantly decreased the proliferation and migration as well colony formation ability of OSCC. [13] MIF-KD substantially negatively affected SCCVII cells proliferation compared to the control. [38] And partial CD74 deficiency resulted to impair proliferative activity together with increasing in the G0/G1 phase of the cell cycle in SCCVII cells, which was associated with lower activation of signaling cascades such as the ERK1/2 MAPK cascade. [12] MIF inhibitor 4-iodo-6-phenylpyrimidine(4-IPP) exerted an inhibitory effect on the proliferation of SCCVII cells in a dose-dependent manner. [81] CPSI-1306, MIF antagonist, could enhance keratinocyte apoptosis and inhibit the UVB-induced epidermal proliferation by promoting p53 degradation, which antagonized UVB-induced squamous carcinogenesis. [82] Xia et al. indicated that MIF exerted a role in protecting bone marrow-derived mesenchymal stem cells from apoptosis *via* the AMPK/mTOR signaling pathway. [83] In malignant pleural mesothelioma cell lines, activated MIF/CD74 pathway had protumorigenic function by increasing tumor cell proliferation and protecting them from apoptosis. [84] Park et al. demonstrated that the combined induction of TAp63 with blockade of the MIF/CD74 signaling pathway could boost apoptosis of malignant B cells. [85] Liu et al. used siRNA to knockdown MIF resulting in proliferation suppression and G0/G1 cell cycle arrest in HEK293 cells. To elucidate the molecular mechanism underlying this phenomenon, they analyzed the genomewide expression profile in MIF-KD cells and normal cells. The results demonstrated that in MIF deficient cells, the positive regulators of G1/S cell cycle progression, Cyclin, CDK, CAK and APC/C were downregulated. However, members of CKI family (p21Cip1/Waf1, p27Kip1 and p57Kip2) antagonizing both cyclin and CDK subunits to block of G1/S transition, were upregulated. It was thought to be related to the inhibition of MAPK, PI3K/Akt, NF-κB, c-Myc-dependent pathways and activation of TGF-β, p53-dependent pathway. [86]

*p53*, one crucial tumor suppressors, is the most commonly silenced or mutated gene in cancer. [87] Generally, p53 level is low or even undetectable, while the cellular p53 protein level rises dramatically in response to the stress signals such as DNA damage, oncogene activation and hypoxia. Then, activated p53 results in a variety of genes activation and transcription which play important roles in cell cycle arrest, senescence, apoptosis, and differentiation. The process ensures that an abnormal cell fails to proliferate, thereby providing a critical barrier against tumor development. [88–90] Hudson et al. pointed out that MIF bypassed the p53-mediated growth arrest or apoptosis with functional screens. Endogenous expressed or exogenously added recombinant MIF was able to inhibit p53-dependent transcriptional activity of p21, cyclin G1, and Mdm2. [91] Subsequently, considerable studies identified MIF as an effective p53 antagonist by inhibiting p53-dependent apoptosis and tumor suppressor role. [92–94] However, the mechanism how MIF achieved this was not yet elucidated. It was speculated that oxidoreductase activity of MIF should be responsible for this inhibition. [95] And Jab1 was deemed to participate in this process. Observations suggested that the ternary MIF-Jab1-p53 complex indeed was a molecular basis for the MIF mediated suppression of the p53-dependent apoptosis. [96, 97] Besides, in the inflammatory microenvironment, NO mediated apoptosis in macrophages *via* p53-dependent manner. [98] Reciprocally, high MIF concentrations sustained monocyte and macrophage function in the face of NO induction of p53-dependent apoptosis. [99]

Autophagy is another manner that manipulates the survival and death of cell. Since it's essential in the degradation of accumulated damaged or long-lived and superfluous organelles which are toxic for cells with the lysosomal machinery, autophagy has been characterized as an intracellular catabolic pathway that plays vital role in maintenance cellular homeostasis. [100] In general, there are three main forms of autophagy in mammalians: microautophagy, chaperone-mediated autophagy (CMA), and macroautophagy, and the latter one is the major subtype of autophagy. The main feature of macroautophagy is the formation of “autophagosomes” in which cytosolic components are sequestrated by plasma membrane to delivered to the lysosome for their breakdown. [101, 102] For the hostile microenvironment such as hypoxia and nutrient depletion in cancer, autophagy can be activated in tumor cells. So far, whether autophagy is beneficial for tumor cell survival or death has been a controversial topic. On the one hand, autophagy endows cancer cells the capability to limit damage and sustain viability by recycling the damaged proteins and organelles. On the other hand, recent studies have discovered that defective autophagy links to increased tumorigenesis. The loss of the essential autophagy gene *beclin1* induced hepatocellular carcinoma, lung adenocarcinoma, mammary hyperplasia, and lymphoma in the mice. The constitutive activation of PI3k/Akt pathway by mutations might stimulate the process. Tamoxifen, used to treat certain types of breast cancer, was considered to activate autophagy by up-regulation *beclin1*. Thus, autophagy is considered as a double-edged sword for its role may be altered during tumor progression. [103, 104]

MIF-induced cytokines, such as IL-1β, TNF-α, have been confirmed to be involved in autophagy. Chuang et al. found that MIF could induce autophagy in hepatocytes through ROS generation. [105] Chen et al. demonstrated that MIF-induced autophagy in endothelial cells caused an increase in vascular permeability. The inhibition of autophagic flux could reverse MIF-induced vascular leakage in both *in vitro* cell culture and *in vivo* mice experiments. [106] In breast cancer cells, Wu et al. revealed that the suppression of MIF increased microtubule associated protein 1 light chain 3 expression, which was in proportion to autophagic vacuole formation and used to quantify autophagy. In the same study, MIF-knockdown enhanced chemosensitivity and suppressed tumorigenesis in the mice by inducing autophagy. [107] In Liu et al. study, MIF inhibition induced ROS-mediated autophagy to rescue cell death in osteosarcoma. [108]

**Figure 1:**
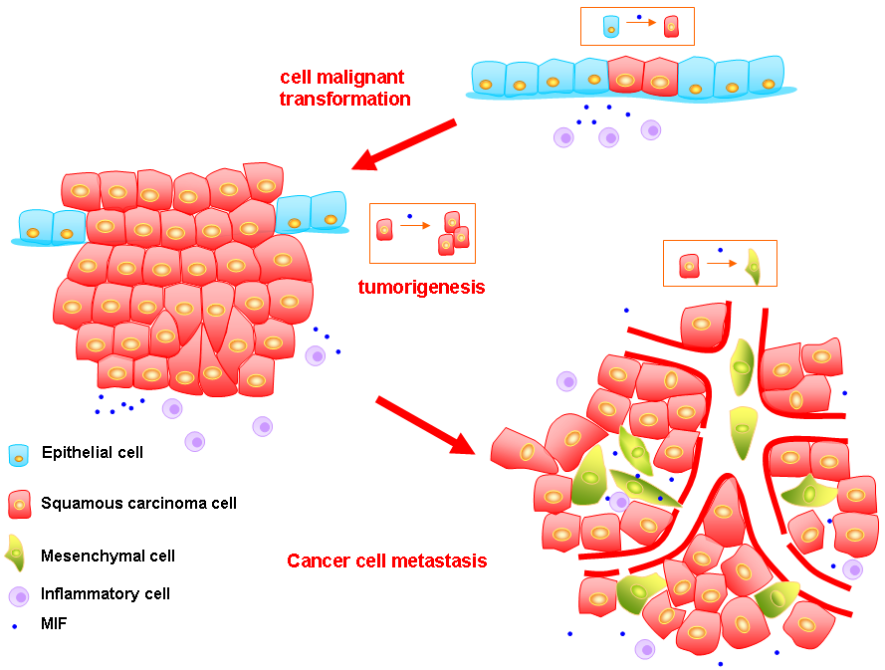
The model of how MIF promoting the progression of HNSCC First, in recurrent or persistent inflammation microenvironment, MIF can promote epithelial cells to undergo malignant transformation. Second, the MIF can increase the cancer cells proliferative activity and inhibit their apoptosis which lead to the formation of primary tumor. MIF also enhances the invasion ability of cancer cells. Meanwhile, the interaction between MIF and HIF-1 is vital to maintain the tumor growth in hypoxia microenvironment. Third, MIF can promote the angiogenesis in HNSCC *via* VEGR and IL-8. However, these vessels are abnormal. Besides, MIF can induce EMT of tumor cells, and these mesenchymal cells are prone to enter the leaky vessels, which fuel the tumor metastasis.

Taken together, the relationship beteewn MIF and HNSCC has been established by a great deal of studies, however, the mechanism is still ambiguous and further research is needed. We summarize the relationship between them mentioned above in the Figure 1.

## A POTENTIAL BIOMARKER FOR HNSCC

With the analysis of quantitative immunohistochemistry, Kindt et al. found that MIF staining intensity significantly increased in hypopharyngeal squamous cell carcinoma samples compared to tumor-free epithelia, low-grade dysplasia or high-grade dysplasia. [109] They had similar results in the laryngeal carcinoma and oral cavity carcinoma. Interestingly, HPV positive oral cavity carcinoma samples exhibited lower level of MIF expression. They further searched whether the MIF expression was correlation with the clinical outcome. Finally, in laryngeal carcinoma, elevated MIF expression was associated with a worse prognosis in terms of local recurrence and cancer metastasis. Reciprocally, in oral cavity carcinoma, it was not correlation with recurrence, while in parallel with the development of a second primary tumor during the follow-up period. [38, 110] Complementally, the serum MIF level of HNSCC patients reached approximately three times compared to their healthy counterparts. [110] By immunostaining MIF in specimens from 50 HNSCC patients treated with chemoradiotherapy, Suzuki et al. demonstrated that MIF-negative was association with poor prognoses. [111] Souza et al. indicated that serological MIF concentration elevated prior to treatment and significantly reduced after tumor resection in oral squamous cell carcinoma patients. [112] High MIF expression in tumor cells were significantly associated with worse prognosis of NPC patients. [113] Thus, MIF can be treated as a potential biomarker for HNSCC. In C3H/HeN mice inoculated orthotopically with MIF-KD or control SCCVII cells, the MIF-KD tumors grew more slowly and appeared more sensitive to Cisplatin, 5-fluorouracil and Taxol. [38] Chang et al. used tricine-SDS-gel-assisted fractionation in conjunction with liquid chromatography-tandem mass spectrometry (LC-MS/MS) to systematically identify low-molecular-weight proteins in the secretomes of five OSCC cell lines and found that MIF was specifically overexpressed in OSCC tumor cells compared to the normal oral epithelium. The overexpression of MIF was associated with cervical metastasis, perineural invasion, deeper tumor invasion, higher overall stage, and a poorer prognosis. Besides, MIF promoted the migration and invasion of OSCC cell lines *in vitro*. Collectively, they proposed that MIF could be a potential tissue biomarker for OSCC. [114] Liu et al. demonstrated that miR-451 downregulated in NPC cell lines and tissue samples leading to enhanced cell migration and invasion *in vitro* and xenograft tumor growth *in vivo* by targeting MIF. [115] In laryngeal carcinoma, the high level of AHNAK combination with MIF up-expression was strongly associated with poor survival. [116]

## CONCLUSIONS

Firstly, MIF was ascribed as a proinflammatory factor. Further studies have demonstrated that MIF is involved in the progression of tumors including HNSCC. The expression of MIF in HNSCC samples has been proved to be related to the clinical outcomes of patients. The higher MIF level in serum was also found in patients with HNSCC. MIF can regulate HNSCC cells proliferation, apoptosis, invasion, and metastasis though its pleiotropic roles in mediating hypoxia response, angiogenesis, and EMT. The inhibition of MIF can restrict the progression of tumors. This evidence hints that MIF may be a potential biomarker of HNSCC.

As mentioned above, for the roles of MIF in supporting cancer, there exists possibility that it can be a therapeutic target for cancer. Indeed, the restriction of MIF showed the efficacy of cancer treatment. The inhibition of MIF in gallbladder cancer cell line by ISO-1 and 4-IPP or its specific siRNA led to a decrease in the colony forming ability. [117] ISO-1, the antagonist of MIF, prevented adenoid cystic carcinoma cell line cell growth and impaired the migration and invasion abilities. [118] Zheng et al. showed that ISO-1 could significantly reduce gastric cancer cell proliferation. Besides, CD74, the receptor of MIF, was also a potential therapeutic target. They found that the knockdown of CD74 or using anti-CD74 mAb could achieve the similar results in gastric cancer. [119] Thus, CD74, as a cell membrane protein, may serve as a therapeutic target, whereas MIF may be viewed as a diagnostic marker.
